# Single cell RNA sequencing analysis and molecular biology experiments identify NAFLD-related fibroblasts and highlights seven hub genes in NAFLD

**DOI:** 10.1371/journal.pone.0332881

**Published:** 2026-02-02

**Authors:** Xuechun Zhang, Ying Cheng, Runzhi Yu, Xiaona Hu, Yiqin Huang

**Affiliations:** 1 Department of Gastroenterology, Huadong Hospital Affiliated to Fudan University, Shanghai, China; 2 Shanghai Key Laboratory of Clinical Geriatric Medicine, Huadong Hospital, Shanghai, China; 3 Department of General Practice, Huadong Hospital Affiliated to Fudan University, Shanghai, China; Helwan University, EGYPT

## Abstract

**Background:**

The Nonalcoholic fatty liver disease (NAFLD) is a major chronic liver condition, with its pathology remaining elusive. Consequently, a thorough exploration of the underlying mechanisms driving NAFLD progression is essential for a comprehensive understanding of this metabolism-associated disorder.

**Methods:**

The scRNA-seq data from liver specimens of healthy and NAFLD subjects from GSE182365 was examined in the current study. High-dimensional weighted gene co-expression network analysis and seven machine learning (ML) approaches were employed to identify and confirm a genetic profile that precisely detects this population. Subsequently, we utilized MCP counter and non-negative matrix factorization to explore immune cell infiltration patterns. Lastly, the comparative expression of seven optimal feature genes *in vivo* and *in vitro* were confirmed through wet experiments.

**Results:**

Single-cell sequencing findings unveiled a distinct fibroblast subset, differentially infiltrated in NAFLD and control cohorts, expressing genes linked to NAFLD pathogenesis. HdWGCNA and ML algorithms screened and identified the essential signature defining this cohort, comprising 7 genes, with function enrichment analysis indicating their strong link to the cell cycle pathway. Notably, we established a connection between these 7 genes and immune cell infiltration and patient variability. Concurrently, wet experiments illustrated the actual expression disparities of our seven genes in NAFLD and normal models both *in vivo* and *in vitro*.

**Conclusions:**

Our investigation has uncovered a distinct fibroblast population closely associated with NAFLD pathogenesis. The related genes, including CCL2, CRYAB, HSBP1, IFIT3, PSMB10, RBX1, and SPARC, with potential promoting or inhibitory roles in NAFLD pathogenesis, were subsequently identified and represented a crucial step in developing prospective clinical diagnostic method for this condition.

## Introduction

The prevalence of metabolic disorders, encompassing nonalcoholic fatty liver disease (NAFLD), exemplified by obesity, has grown exponentially in the recent decades [1]. A cohort of expert consensus recently proposed that the designation metabolic dysfunction-associated fatty liver disease (MAFLD) might be utilized to more precisely describe the primary metabolic aspect linked to hepatic steatosis [2]. NAFLD encompasses a range from basic liver fat buildup (hepatic steatosis) to nonalcoholic steatohepatitis (NASH), which can advance to liver fibrosis, cirrhosis, and hepatocellular carcinomas [3]. NAFLD is closely linked to metabolic syndrome, including dyslipidemia, hypertension, obesity and type 2 diabetes mellitus (T2DM) [4]. Current understanding suggests that the pathophysiology of this condition primarily involves metabolic dysfunction and lipotoxicity; however, the reality is more complex, involving nutritional factors and lifestyle, various genetic variations, altered gut microbiome, dysregulated innate and adaptive immunity, and fibroblast activation [5].

Over the past ten years, individual cell RNA sequencing has risen as an emerging research tool, the analysis of which is widely used in all kinds of diseases.

The MiRNAs are known to modulate target genes expression at post-transcriptional levels, through reduction of spatial abundance of mRNA. The recent technologies have focused on computational biology and biotechnology. The modulation of MiRNA has been possible through recently available biotechnological tools such as CRISPR Cas9, TALENS and RNAi). In the recent era of technology, [6] The Machine learning (ML) an artificial intelligence method has been widely performed to discover markers for new diagnostic and prognostic biomarkers. It offers several advantages such as low fault, wider applications and has the ability to handle multidimensional data. [7]The current study was utilized together with R language to discover the difference in cell clusters between NAFLD and control cohorts, which helped us to focus our research on fibroblasts. Then, combined with conventional bulk RNA-seq analysis and machine learning (ML) methods, we further explored the key genes and their relationship with immune infiltration in the NAFLD dataset. Finally, the hypothetical molecular pathways through which these crucial genes influence the development of NAFLD were preliminarily identified.

## Methodologies and materials

### Data acquisition

The samples included one gene expression data base from musmusculus and 5 samples from homosapiens. The scRNA-seq GSE182365 was acquired from the Gene Expression Omnibus (GEO) database (https://www.ncbi. nlm.nih.gov/geo/) and processed with the conventional Seurat pipeline. RNA-seq bulk datasets, GSE63067, GSE89632, GSE48452, GSE66676, and GSE135251, which were also from the GEO database, were annotated with the official platform. Comprehensive information about the datasets and patient metadata, as depicted in the original publications, has been summarized in (Table 1).

**Table 1:** Summary of key characteristics for the examined dataset on animal and humans.

Dataset	Year	Organism	Case	Control	Sample Source	Avg. Cells per Sample	Description	Platform	Datatype
**GSE182365**	2021	Mus musculus	1	1	liver cells	~20,000	Single cell RNA-seq of liver cells from B6J mice fed a chow or a high-fat-and-high-sucrose diet	GPL19057Illumina NextSeq 500	scRNA-seq
**GSE63067**	2014	Homo sapiens	9	7	liver samples	N/A	Human liver samples of steatosis and non-alcoholic steatohepatitis were selected for RNA extraction and hybridization on Affymetrix microarrays.	GPL570 [HG-U133_Plus_2] Affymetrix Human Genome U133 Plus 2.0 Array	Microarray
**GSE89632**	2016	Homo sapiens	19	24	living liver	N/A	Genome-wide analysis of hepatic gene expression in patients with non-alcoholic fatty liver disease and in healthy donors in relation to hepatic fatty acid composition and other nutritional factors	GPL14951Illumina HumanHT-12 WG-DASL V4.0 R2 expression beadchip	Microarray
**GSE48452**	2013	Homo sapiens	32	18	liver biopsy	N/A	Human liver biopsy of 73 samples grouped into C (control = 14), H (healthy obese = 27), S (steatosis = 14) and N (nash = 18).	GPL11532 [HuGene-1_1-st] Affymetrix Human Gene 1.1 ST Array [transcript (gene) version]	Microarray
**GSE66676**	2017	Homo sapiens	26	33	liver biopsies	N/A	Prospective observational cohort study, where liver biopsies were obtain intra-operatively and submitted for Human Gene St 1 microarray.	GPL6244 [HuGene-1_0-st] Affymetrix Human Gene 1.0 ST Array [transcript (gene) version]	Microarray
**GSE135251**	2020	Homo sapiens	206	10	frozen liver biopsies	N/A	206 NAFLD cases and 10 contrls snap-frozen biopsies were processed for RNA sequencing on the Illumina NextSeq 500 system.	GPL18573Illumina NextSeq 500 (Homo sapiens)	Bulk RNA-seq

### Single‑cell sequencing data processing

ScRNA-seq data from GSE182365 was examined utilizing the Seurat R package (Version 3.2.0). During the quality control phase, gene reads per cell were constrained between 200 and 2500. The mitochondrial gene proportion filtering threshold was established at 10%. We employed the “FindVariableFeature” function within Seurat to detect highly variable genes [8]. Principal component analysis was executed, and the cells were subsequently projected and visualized in 2D space using Uniform Manifold Approximation and Projection (UMAP). Ultimately, we manually classified cell types based on established cell markers [9].

### Cell–cell interaction analysis

Cell to cell interaction was inferred utilizing the CellChat R package (version 1.0.0) to depict the intercellular signaling networks among all cell clusters and investigate the ligand-receptor-mediated interactions between them [10]. Subsequently, we employed the net visual circle and visual bubble functions to graphically represent the magnitude and strength of cellular communication from the particular target cluster to the other cell cohorts. The primary signal inputs and outputs across all cell subpopulations were evaluated using CellChatDB.Human on cell chat [11].

### Pseudo time analysis

Pseudo-temporal analysis was conducted utilizing the R package “Monocle2” with its default parameters. Cellular differentiation states were ascertained through the DDR tree algorithm and the “reduce Dimension” function in the Monocle toolkit, succeeded by employing the “plot_cell_trajectory” function to render the cellular differentiation path [12]. Subsequently, the impacted genes were identified from the top 50 markers across clusters using the Differential Gene Test function and were visualized via the plot_pseudotime_heatmap function. The num_cluster parameter was configured to 6 to yield six modules of markedly altered genes exhibiting comparable trends based on their pseudo-temporal expression profiles.

### High dimensional WGCNA (hdWCNA)

The process of building co-expression networks and detecting stable clusters of associated genes in R was executed using the “hdWGCNA” package [13]. During the preliminary stage, the gene expression dataset was subjected to preprocessing in order to eradicate noise and mitigate batch effects. Next, we established a gene co-expression network grounded in pairwise gene correlations. Within this network, we identified clusters of highly interrelated genes and computed the module eigengenes. Subsequently, we conducted module preservation analysis to evaluate the stability of the detected clusters and pinpoint feature genes (FGs) of the NAFLD-associated modules. The final phase involved s performing functional enrichment analysis to uncover the biological processes and pathways connected to these modules.

### Construction of ML model

To pinpoint crucial diagnostic genes, we employed univariate logistic regression, followed by variable refinement utilizing the Least Absolute Shrinkage and Selection Operator (LASSO) technique [14]. To construct models exhibiting superior accuracy and resilient performance, we utilized the “mlr3” R package for model creation [15]. We incorporated seven ML algorithms, namely k-nearest neighbor (KNN), linear discriminant analysis (LDA), logistic regression (LR), Naïve Bayes (NB), random forest (Ranger), Recursive Partitioning and Regression Trees (RPART), and Support Vector Machine (SVM), to determine the most effective model [16].

### Immune infiltration analysis

CIBERSORTx is an instrument that employs a collection of gene expression signature matrices from pure cell populations to approximate the relative fractions of various cell types within a given specimen [17]. The CIBERSORT methodology was applied to detect the infiltration of 10 immune cell types in both normal and NAFLD specimens. The MCP counter algorithm, implemented through the R package “MCPcounter”,was utilized to assess the prevalence of 10 cell populations [18,19]. These findings were subsequently visualized and statistically examined using the R package “ggplot2”. Box plots were employed to depict the immune cell makeup of individuals exhibiting different immune patterns. The Wilcoxon rank-sum test was conducted to assess the disparities in immune cell fractions, with P < 0.05,which was deemed statistically significant.

### RNA‑seq bulk NMF

The NMF examination was executed utilizing the R package “NMF” [17].The optimal quantity of clusters were selected according to cophenetic coefficients. This procedure encompassed applying the NMF model to the preprocessed dataset and deriving the factor matrices, which revealed the importance of individual variables within each factor and the influence of each factor on every specimen. The grouping outcomes of NAFLD subjects were visually depicted employing the “consensus map” function.

### Differential expression analysis

Differential expression analyses between NAFLD and control specimens in selected GEO datasets were performed to detect DEGs utilizing the R package “limma” (parameters: | logFC| > 0.75, P-value < 0.05). The outcomes were illustrated via box plot.

### Single sample gene set enrichment analysis (ssGSEA) and gene set enrichment analysis (GSEA)

To delve into the functional activation of our target gene sets and elucidate the biological relevance of key FGs, we employed the ssGSEA algorithm from the GSVA package (version 1.42.0) [20] alongside the GSEA workflow, which offers comprehensive insights into relevant pathways [21]. We performed a gene set permutation 1,000 times for every analysis inorder to derive a normalized enrichment score. Substantial enrichment was established using an FDR < 0.05.

### Cell culture and treatment

The LX2 cell lines were procured from the Shanghai Institute for Biological Science (China). These cells were kept in DMEM enriched with 10% FBS, 100 U/ml penicillin, and 100 μg/ml streptomycin (Invitrogen, CA, USA) at 37 °C in a moisture-controlled incubator with 5% CO_2_. For experimental treatments, a FFAs mixture was formulated using oleic acid (OA; Sigma, O1257) and palmitic acid (PA; Sigma, P0500-10G) in a 2:1 proportion and was applied at a 0.5 mmol/L final concentration for 24 hours to induce excessive FFAs uptake.

### Animal study (methods of sacrifice and pain alleivation) and tissue staining

Male C57BL/6J mice (8–10 weeks old, healthy) were obtained from a certified supplier and housed in a specific pathogen-free (SPF) facility under controlled conditions (temperature: 22–24°C, humidity: 55%–60%, 12-hour light/dark cycle). Mice were randomly assigned to two groups: the NAFLD group received a high-fat diet (Research Diets, Inc.) for 16 weeks, while control mice were provided standard chow and water ad libitum. All animal procedures were reviewed and approved by the Institutional Animal Care and Use Committee of Huadong Hospital, Fudan University (Approval No. 2023K093), and carried out in accordance with national regulations on laboratory animal care and use.

Animal well-being were monitored daily. Humane endpoints were predefined, including signs such as severe weakness, weight loss exceeding 20%, or markedly reduced activity. At the experimental endpoint, mice were anesthetized with 1.5%–2.0% isoflurane and euthanized by cervical dislocation, a method consistent with AVMA guidelines. All procedures were performed by trained personnel to ensure minimal discomfort.

For Oil Red O staining, liver tissues were embedded in optimal cutting temperature (OCT) compound, cryosectioned at 10 μm thickness, and stained with Oil Red O (Sigma-Aldrich) to visualize lipid accumulation. For hematoxylin and eosin (H&E) staining, liver tissues were fixed in 4% paraformaldehyde overnight, paraffin-embedded, sectioned at 5 μm, and stained using standard protocols. Hepatic triglyceride (TG) and total cholesterol (TC) levels were measured using commercial assay kits (Njjcbio) according to the manufacturer’s instructions.

Male C57BL/6J mice, aged 8–10 weeks, with good health, were maintained in enclosures under typical lab settings (temperature range of 22–24°C, 55%–60% relative humidity, and alternating 12-hour light/dark periods). A NAFLD mouse model was created by administering a high-fat diet (obtained from Research Diets, Inc.) for 16 weeks. The control cohort received standard chow and water.

### Quantitative real-time PCR

Hepatic tissue RNAs were isolated utilizing TRIzol reagent (Invitrogen) and subsequently converted to cDNA utilizing the First Strand cDNA Synthesis Kit (Takara), as per the provided protocol. Gene transcript quantification was conducted via quantitative real-time PCR employing SYBR Green Premix Ex Taq (Takara). Gene expression data analysis utilized the 2^−ΔΔCt^ approach for comparative quantitation. Expression results were standardized against GAPDH mRNA levels. Table 2 depicts the primer sequences used.

**Table 2:** Primers for all 7-genes ascertained by ML algorithms for qPCR analysis.

Gene	Forward primer (5’-3’)	Reversed primer (5’-3’)
*Ccl2*	GCTACAAGAGGATCACCAGCAG	CTTTTTGCTTGTTGTTGGTCTCC
*Cryab*	CGGAGGAACTCAAAGTCAAGGTT	ATGGTGAGAGGATCCACATCGG
*Hsbp1*	GTCAGACCAGATCATCGGAAGG	GGTCCAGTTCTTCTACTCCAGC
*Ifit3*	GCTCAGGCTTACGTTGACAAGG	CTTTAGGCGTGTCCATCCTTCC
*Psmb10*	TTCGGGTTCCTCATGCACGCAA	TTTGTCCGCCACAACCGAATCG
*Rbx1*	GTGGTTGATAACTGTGCCATCTG	CATGCAACCGTACACTCTTCGG
*Sparc*	CACCTGGACTACATCGGACCAT	CTGCTTCTCAGTGAGGAGGTTG

### Western blots (WBs)

WB was executed to examine the related protein activities in the liver. Hepatic homogenate was re suspended in radioimmunoprecipitation (RIPA) buffer containing protease inhibitors cocktail (Beyotime) to obtain total protein extracts. Protein quantification was performed using BCA kits (EpiZyme), and equal quantities of protein were resolved on a 10% SDS-PAGE gel before being moved to a PVDF membrane. The membranes underwent blocking in 5% milk solution and were incubated with primary antibodies overnight at 4°C. Following incubation with secondary antibodies for 60 minutes at ambient temperature, signals were visualized utilizing the Odyssey imaging system.The GAPDH served as the internal control.

### Statistical analysis

All data processing, statistical examination, and visualization were performed using R software (version 4.1.3) and GraphPad Prism (version 9.5). The investigation utilized the Wilcoxon test for comparisons between two cohorts, while the Kruskal–Wallis test was applied for three-group analyses. Pearson correlation analysis was employed to investigate the relationship between 7 essential genes and 10 immune cell categories. All statistical P-values were two-tailed, and P < 0.05 was deemed statistically significant.

## Results

### Single cell RNA-sequencing analysis of NAFLD

**Fig 1:**
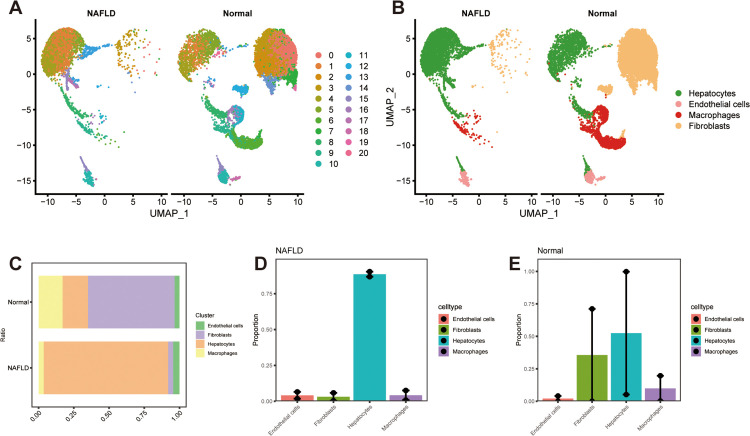
UMAP visualization of single-cell transcriptomic data from NAFLD and normal liver samples. (A) UMAP plot shows clustering of distinct liver cell types. (B) Cells were manually annotated based on marker genes. Gene expression values were log2-normalized and scaled using the Seurat pipeline. (C) Cluster distribution in NAFLD vs. normal samples. Statistical significance was determined using the Wilcoxon rank-sum test (P < 0.05 was considered significant). (D) Bar plot indicates the cell proportion of all four cell types in NAFLD livers. (E) Bar plot indicates the cell proportion of all four cell types in normal livers.

In NAFLD liver, numerous alterations occur functionally as well in the cellular ratios therefore, we investigated how liver is altered in the overall setting of NAFLD. Hence, we procured scRNA-seq data from GSE182365 comprising mouse liver cells from normal and NAFLD cohorts. Substandard cells were eliminated by the standard of nFeature_RNA > 200 & nFeature_RNA < 2500 & percentmt<10. Exultingly, the number of cells were narrowed from 29,955–15,704 (S1A and S1B Fig). After performing a HARMONY analysis to eliminate batch effect between samples, which went through PCA dimensionality reduction analysis (S1C–S1E Fig), we obtained 20 subtypes by UMAP plot visualization (Fig 1A). Cell populations were manually classified, resulting in four cell types: hepatocytes, endothelial cells, macrophages, and fibroblasts (Fig 1B). The distribution of various cell types has been demonstrated in Fig 1C–1E. Notably, the percentage of fibroblasts is much higher in normal liver than in NAFLD (Fig 1C–1E), indicating it is a part of the evolution of NAFLD.

**Fig 2:**
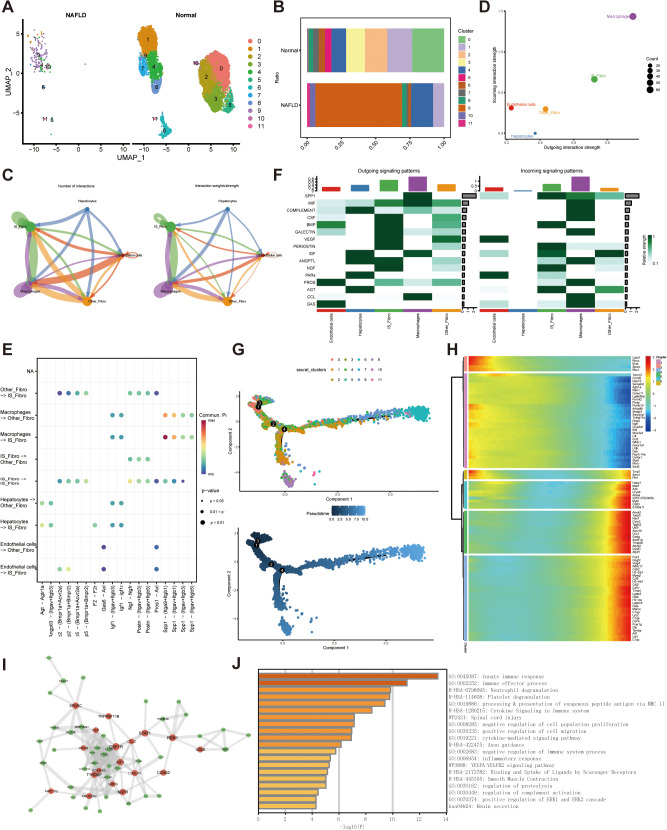
Single cell analysis of cell proportion of fibroblasts and cell-cell communications between the cell types. (A) UMAP plot depicts the arrangement of diverse subclusters of fibroblasts in NAFLD and normal livers. (B) CP of 11 fibroblast subtypes in NAFLD and normal livers. (C) Cellchat analysis of “IS_Fibro”, “Other_Fibro” and other cell types. (D) Scatter plot highlights the variations in incoming and outgoing interaction intensities across various cell categories. (E) Bubble plots depicted the relevant signaling cascades recorded in the “CellChat” R package in cell–cell communications. (F) Signaling function assessment on the consolidated cell-cell interaction network from all signaling cascades among fibroblast subtypes. (G) The trajectory graph exhibited the developmental phases of fibroblast subpopulations. (H) Pseudotemporal heatmap displaying gene expression dynamics for key marker genes. Genes (rows) were grouped into 6 modules. (I) The co-expression network illustrating the correlation strength of central genes from intersecting candidate genes. (J) Functional enrichment plot executed by Metascape revealed the most statistically significant enriched categories.

Studies indicate that while dormant hepatic stellate cells exhibit relative uniformity, their activated counterparts and myofibroblasts display greater diversity, aligning with our findings [22].Automatically, additional examination utilizing shared nearest-neighbor clustering via the Seurat single-cell R toolkit was conducted on fibroblasts, yielding 12 adjacent sub clusters. The HARMONY technique was employed to mitigate batch effects between samples. (S2A–S2C Fig). Eight subclusters were gained in NAFLD fibroblasts, but 12 in normal fibroblasts (Fig 2A). Therefore, we defined the four exclusive subtypes 0,2,3,5 in normal liver as “IS_Fibro”, and the remaining subtypes, which were expressed both in two cohorts, as “Other_Fibro”. Detailed differences in the proportions can be observed in Fig 2B. To elucidate intercellular interactions and target cell types during NAFLD progression, we utilized the CellChat package (Version 1.0.0) for communication analysis. Complex interactions were found between the cell subtypes (Fig 2C). The results showed that “IS_Fibro” interacts more extensively with other cell types than “OtherFibro” does (Fig 2D). We then explored particular signaling routes where these cellular populations display more pronounced mutual interactions.The outcomes indicated that “IS_Fibro” demonstrated more robust communication, as determined by the “CellChat” package, relative to “Other_Fibro”, transmitting more signals to hepatocytes, macrophages, and endothelial cells. The signaling patterns are richer, too (Fig 2F).

Temporal sequencing of cellular states for these fibroblast subgroups was achieved through pseudotime trajectory analysis. Consequently, “IS_Fibro” (encompassing clusters 0, 2, 4, and 5) was positioned near the trajectory’s starting point (Fig 2G). Additionally, we investigated the pseudotemporal dynamics of genes exhibiting significant changes across these 11 subclusters and categorized them into six modules based on their expression trajectories over pseudotime (Fig 2H).

### HdWGCNA analysis underscores the core modules for characterizing the prospective functions of clusters

**Fig 3:**
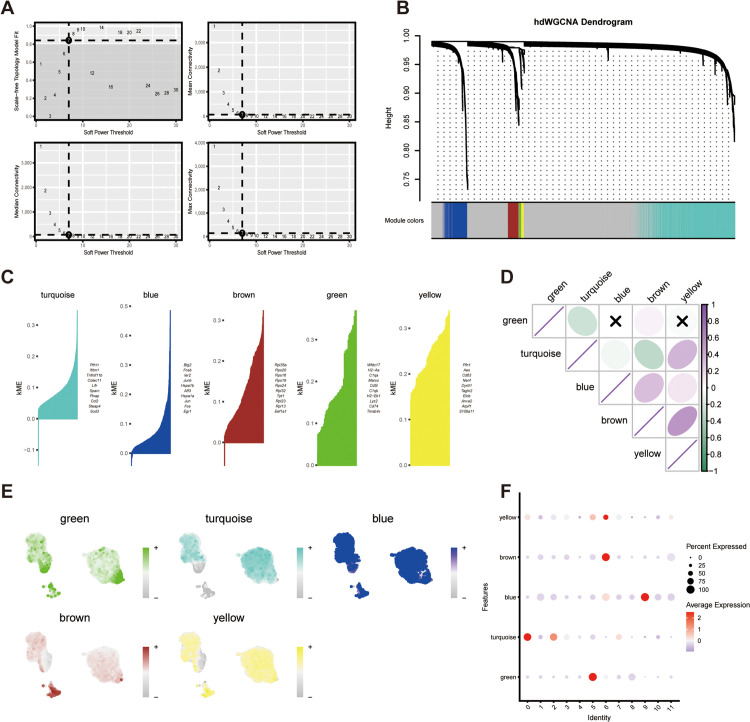
hdWGCNA of fibroblasts unveils the pivotal functions of the turquoise module. (A) The upper-left graph illustrates the soft power threshold for selecting a scale-free topology model. The remaining three panels displayed the average, median, and maximum connectivity of the topological network, respectively, when various minimum soft thresholds are applied, indicating the network’s connectivity. The network’s mean connectivity exhibits the greatest stability at the lowest soft threshold of 7. (B) As demonstrated in the hdWGCNA dendrogram, five modules were identified. (C) Five gene modules were derived, and the leading HGs were depicted in accordance with the hdWGCNA workflow. (D) A correlation analysis was performed on the five modules. (E) Feather plots illustrated the corresponding module scores within fibroblasts. (F) The bubble chart showcased the scores attained by five modules across fibroblast subtypes.

**Fig 4:**
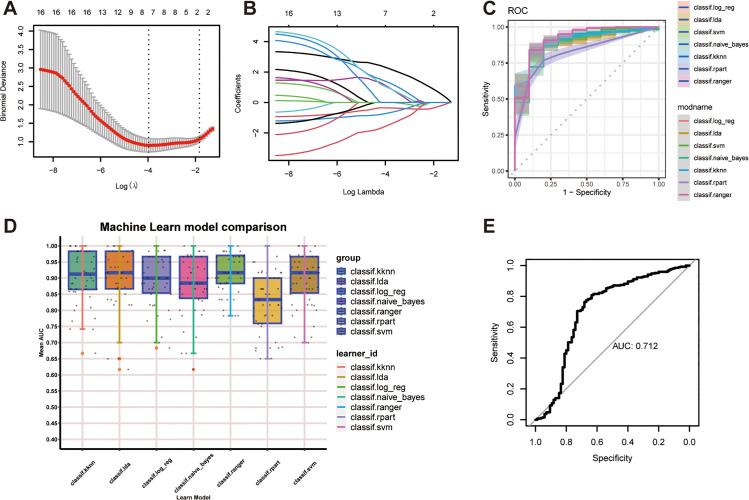
ROC curve analysis of different machine learning models for predicting NAFLD-related gene expression patterns. (A) The LASSO model selected 7 key genes. (B)Coefficient changes of the selected features using lasso algotithm. (C) ROC curves for 7 ML models (KNN, LDA, LR, Naïve Bayes, Random Forest, RPART, and SVM). (https://CRAN.Rproject.org/package=mlr3verse). (D) Receiver operating characteristic values for all seven algorithms displayed in the training cohort. E) ROC values of the “ifRanger” model demonstrated in the test cohor.

We applied a high Dimension WGCNA pipeline to the GSE182365 dataset to investigate co-expressed gene modules and hub genes (HGs) in fibroblasts. The soft threshold was set to 7 (Fig 3A). As shown in Fig 3B and 3C, 5 gene modules were obtained based on the TOM, namely turquoise, blue, brown, green, and yellow modules. And the top huh genes were depicted in accordance with the hdWGCNA pipeline. The positive or negative correlation between the modules was of varying intensity (Fig 3D). Additionally, we evaluated the activation state of each color module in all the fibroblast clusters by the module scores (Fig 3E and 3F). Notably, the turquoise module displayed high activation primarily in “IS_Fibro” cluster (Fig 3F), implying their pivotal role in NAFLD. As specified that the genes within the turquoise module may be of great significance in the distinction between NAFLD and healthy people, we aimed to develop a dependable predictive model for diagnosing NAFLD in gene expression levels. Firstly, we employed the LASSO algorithm to further refine the genes in the turquoise module (Fig 4A and 4B), among which only 7 genes were deemed potent for classifier construction: CCL2, CRYAB, HSBP1, IFIT3, PSMB10, RBX1, SPARC. Furthermore, 5 GEO datasets served as training cohorts (GSE63067, GSE89632) or texting cohorts (GSE48452, GSE66676, GSE135251) and were collected for the subsequent ML training and texting. Altogether, we had 31 healthy people and 50 NAFLD patients (from simple steatosis to steatohepatitis) in the training cohort. While in the testing cohort, we had up to 85 healthy people and 271 NAFLD patients. Thus, the ROC curve was utilized to calculate the accuracy and the performance of seven ML algorithms were estimated accordingly (Fig 4C). AUC data showed that the “ifRanger” potentially exhibited superior efficacy in both internal and external cohorts (Fig 4D and 4E). In conclusion, via “ifRanger” algorithms, we preliminary built a diagnostic binary classification model, trying to classify NAFLD from healthy people from the perspective of these seven genes.

**Fig 5:**
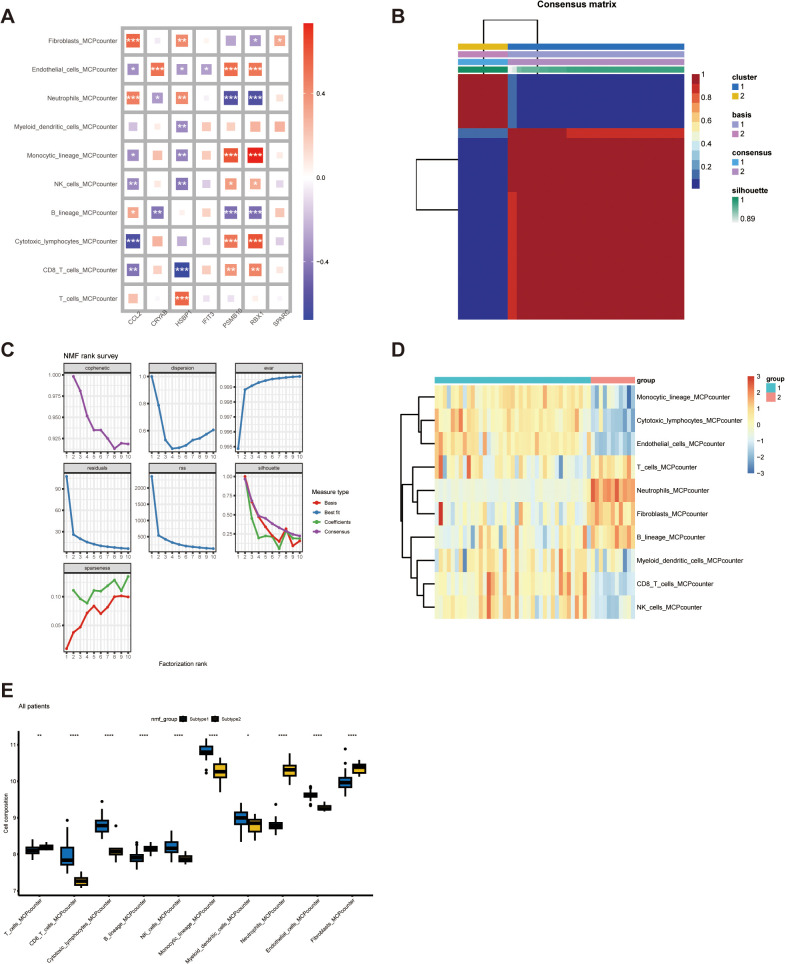
The expression pattern of the 7 genes was linked to immune infiltration and NAFLD patients subtyping. (A) The correlation matrix visualized the relationships between the expression levels of 7 genes identified through LASSO regression and various immune cell types. (B) The consensus heatmap illustrated NAFLD patient subgroups when k-means were set to two using NMF techniques. (C) The value of k at which the cophenetic correlation coefficient’s magnitude started to decrease was 2. (D) The heat map juxtaposed the immune cell infiltration patterns across two NAFLD subtypes as determined by the MCP counter. (E) The box plot contrasted the immune cell infiltration patterns between the two NAFLD subtypes.

**Fig 6:**
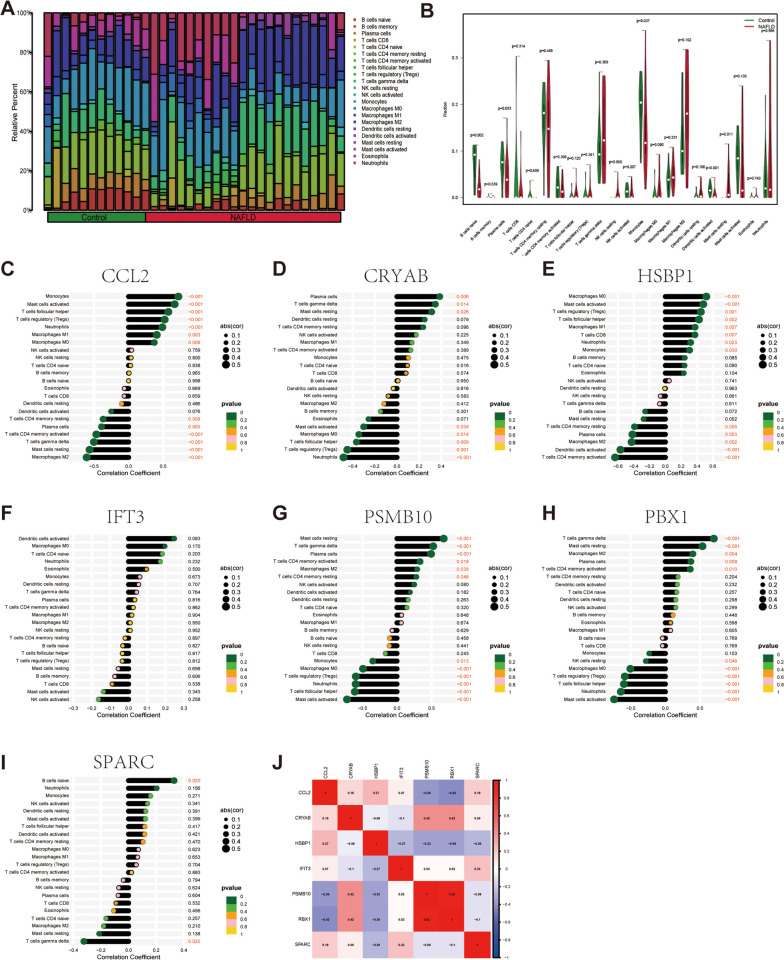
Differential immune cell infiltration between NAFLD and control samples. (A) Bar plots show relative proportions of 22 immune cell types in control and NAFLD samples. (B) Statistical significance was assessed using the Wilcoxon rank-sum test, with multiple testing correction via the Benjamini-Hochberg method (FDR < 0.05). Correlation between immune cells and optimal feature genes CCL2 (C), CRYAB (D), HSBP1 (E), IFT3 (F), PSMB10 (G), PBX1 (H), and SPARC (I). (J) Correlation analysis of seven optimal feature genes in NAFLD samples.

Various immune cell populations in NAFLD constitute the immune landscape of damaged liver, which is widely recognized as a key pathogenic mechanism of NAFLD [23]. Therefore, we further investigated the link between our essential genes and the nonparenchymal cells, primarily immune cells, at the bulk RNA-seq level. Initially, we estimated cell component abundance in CD lamina propria by developing scRNA-seq derived personalized signatures using CIBERSORTx. The results indicated that CCL2 displayed a significant positive association with fibroblasts and neutrophils, while showing a marked negative association with cytotoxic lymphocytes. CRYAB demonstrated a marked positive link to endothelial cells; HSBP1 displayed a significant negative association with CD8 + T cells but a positive link to T cells. PSMB10 depicted a significant positive association with monocytic lineage and cytotoxic lymphocytes, while displaying a significant negative association with neutrophils and B cells. The link between RBX1 and immune cells resembled that of PSMB10 and immune cells (Fig 5A). Additionally, a consensus matrix was generated using the NMF algorithm to categorize the samples in the aforesaid datasets into two clusters grounded in their median expression levels of the 7 genes. As illustrated in Fig 5B, the expression pattern of these genes varied markedly in two NAFLD subtypes. Regarding the number of clusters, we chose values of k, where the cophenetic correlation coefficient’s magnitude starts to decrease as our optimal rank number, which is 2 (Fig 5C). Compared to subtype2, the proportions of CD8 + T cells, NK cells, and cytotoxic lymphocytes were markedly elevated in subtype1, while the proportion of B cells, neutrophils, and fibroblasts were markedly reduced (Fig 5D and 5E). Subsequently, we evaluated the disparities in immune cell infiltration between NAFLD and control specimens, utilizing the CIBERSORT algorithm. The findings for differential immune cell infiltration are presented in Fig 6A and 6B. Only the differences in naive B cells, monocytes, activated dendritic cells, and resting mast cells were significant. To extract detailed information from Fig 5, we proceeded with correlation analysis for the immune cell types and the seven FGs. CCL2, HSBP1, PSMB10, and PBX1 exhibited more significant relevance to immune cells. For example, the HSBP1 gene is positively linked to macrophages M0 (P < 0.001) and activated mast cells (P < 0.001) but highly negatively correlated with resting memory CD4 T cells (P < 0.001) and activated dendritic cells (P < 0.001) (Fig 6C–6I). Gene correlations were also investigated, as depicted in Fig 6J. Some of these FGs demonstrated a notable correlation. Notably, the correlation coefficient between PSMB10 and RBX1 was 0.92, suggesting a potential functional similarity.

**Fig 7:**
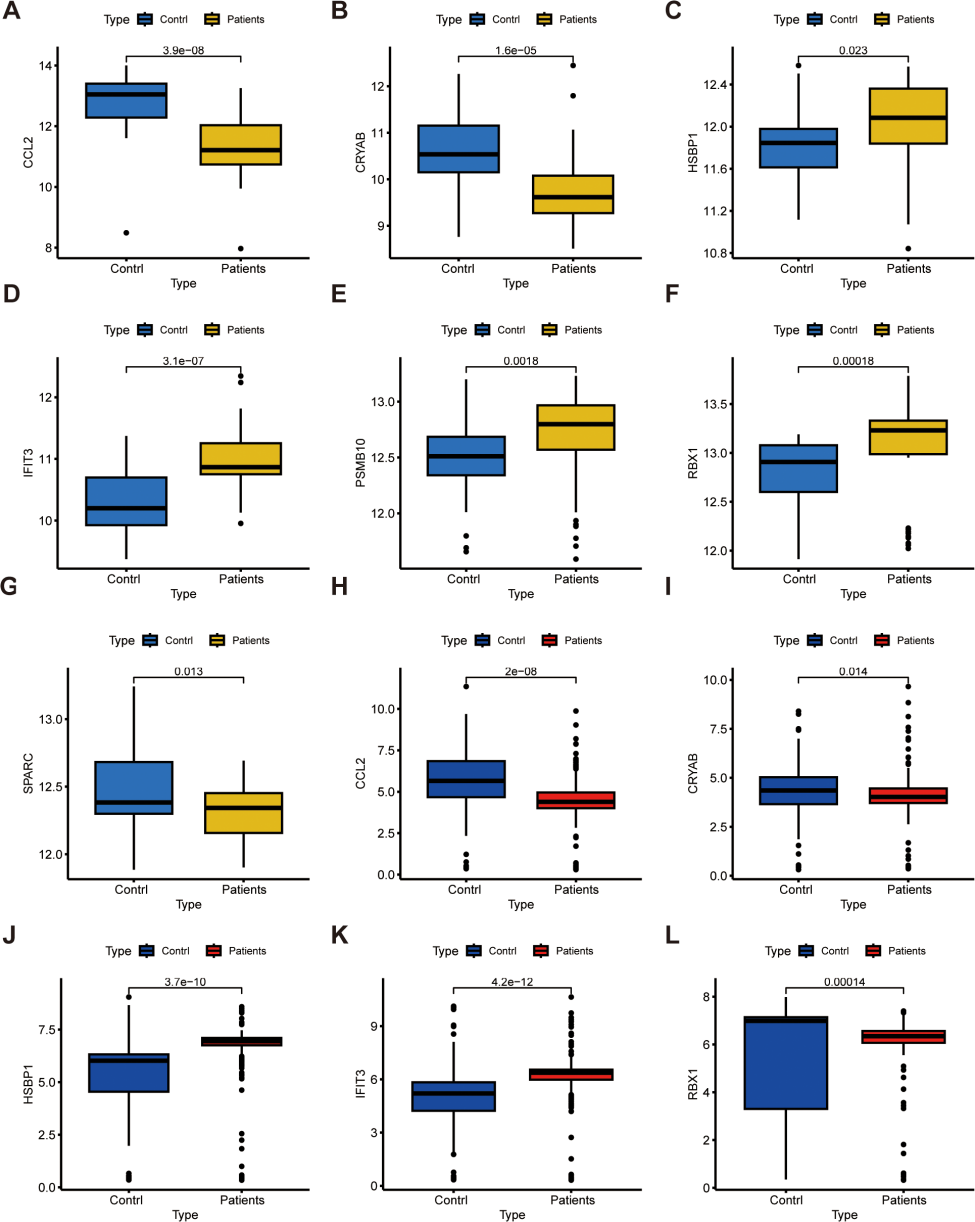
Confirmation of expression for the seven FGs utilizing internal and external validation datasets. (A–G) Box plots depicting the expression of CCL2 (A), CRYAB (B), HSBP1 (C), IFT3 (D), PSMB10 (E), PBX1 (F) and SPARC (G) in control and NAFLD specimens. (H)- CCL2,(I)- CRYAB,(J) -- HSBP1 (K)- IFT3,(L)- PBX1.

We additionally assessed the expression patterns of the 7 key FGs across 50 NAFLD specimens and 31 control specimens. The expression of HSBP1, IFIT3, PSMB10, and RBX1 was notably elevated in NAFLD cohorts, whereas CCL2, CRYAB, and SPARC exhibited reduced expression in NAFLD cohorts, suggesting their potential involvement in NAFLD progression. (Fig 7A–7G), (P < 0.05). Moreover, to enhance accuracy and reliability, we assessed the expression of these key FGs in an external validation dataset comprising 271 NAFLD specimens and 85 control specimens. As illustrated in (Fig 7H–7L), 5 out of 7 key FGs displayed significant expression differences between the two cohorts. The external validation outcomes strongly indicate their high diagnostic potential for NAFLD.

**Fig 8:**
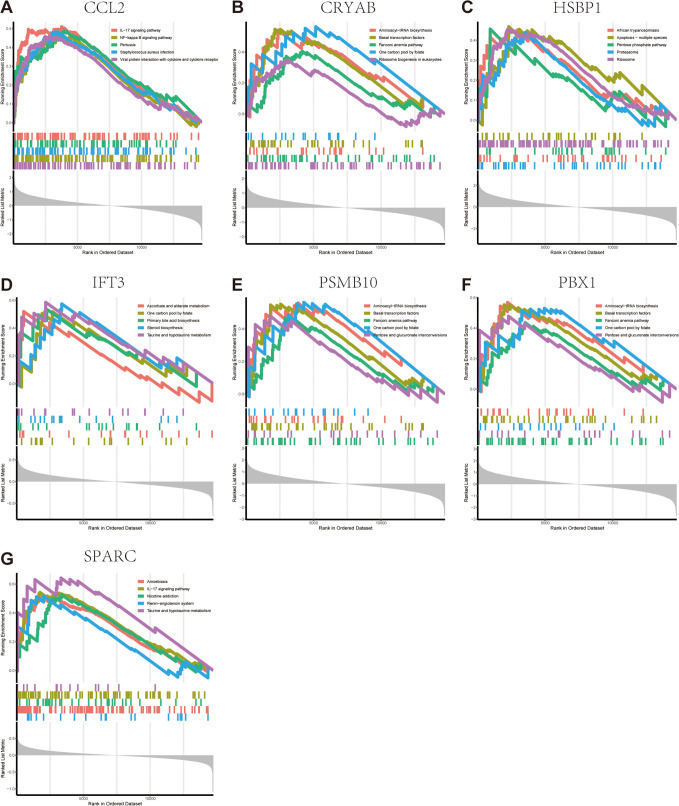
GSEA reveals signaling cascades within the selected FGs. Top five signaling cascades that are markedly enriched in the high expression of CCL2 (A), CRYAB (B), HSBP1 (C), IFT3 (D), PSMB10 (E), PBX1 (F) and SPARC(G).

As these seven FGs possess substantial prognostic value, we subsequently conducted a GSEA analysis to elucidate their potential biological roles. NAFLD samples were categorized into two cohorts grounded in the median expression levels of each FG. The IL-17 signaling cascade, NF-kappa B signaling cascade, pertussis, Staphylococcus aureus infection, and the interaction between viral proteins and cytokines or their receptors (Fig 8A). Elevated CRYAB expression was linked to pathways like Aminoacyl-tRNA and ribosome biosynthesis, basal transcription factors, Fanconi anemia pathway, and One carbon pool by folate (Fig 8B). African trypanosomiasis, apoptosis, pentose phosphate pathway, proteasome, and ribosome were markedly overrepresented in the elevated HSBP1 subset (Fig 8C). Increased IFIT3 expression was linked to pathways including ascorbate and aldarate metabolism, one carbon pool by folate, and primary bile acid biosynthesis (Fig 8D). PSMB10 and PBX1 exhibited similar enrichment patterns with aminoacyl-tRNA biosynthesis, basal transcription factors, Fanconi anemia pathway, one carbon pool by folate, and pentose and glucuronate interconversion (Fig 8E and 8F). Amoebiasis, IL-17 signaling cascade, nicotine dependence, renin-angiotensin system, and taurine and hypotaurine metabolism pathway were markedly overrepresented in the elevated SPARC subset (Fig 8G). The enrichment profile of the low target gene cohorts has been presented in S3 Fig.

**Fig 9:**
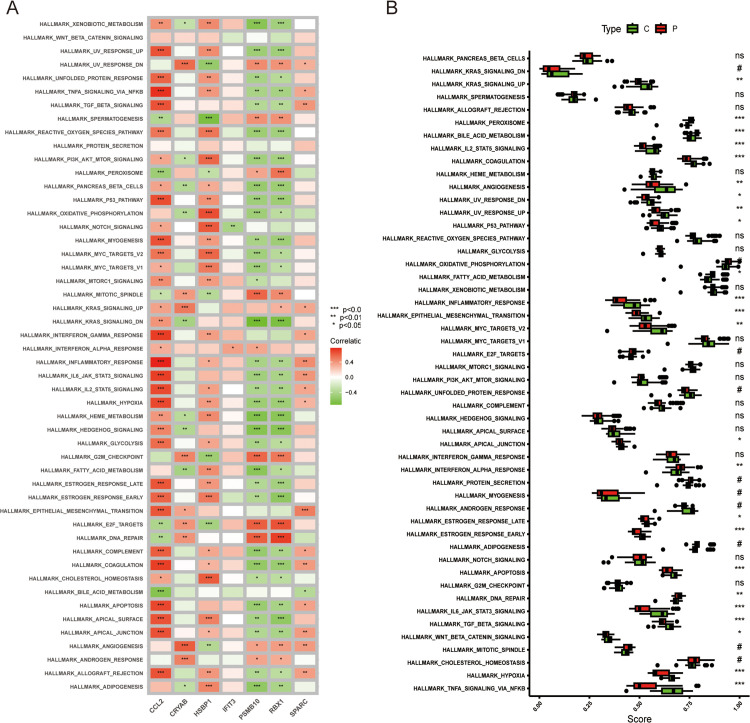
Examination of HGSs. (A) Correlation analysis of the 50 HGSs with seven optimal FGs. (B) The specific allocation of the 50 HGSs in NAFLD and control specimens.

Additionally, to examine whether hallmark gene set (HGS) enrichment varies between the NAFLD and control cohorts, we assessed the significance of disparities across 50 HGSs using the ssGSEA algorithm based on the enrichment scores. The detailed distribution profile is shown in Fig 9A. Several HGSs displayed notable differences, including classic NAFLD-related sets such as genes involved in adipogenesis, unfolded protein response, cholesterol homeostasis, peroxisome, inflammatory response, coagulation, and apoptosis, as well the mitotic spindle, KRAS-signaling-down, E2F_targets, myogenesis, androgen-response, bile acid metabolism, and IL-2_STAT5 signaling. Moreover, the comprehensive GSEA analysis of the 50 HGSs in the 7 genes is represented in Fig 9B. These findings necessitate further in-depth exploration of the diverse functions of the prime FGs in NAFLD development.

**Fig 10:**
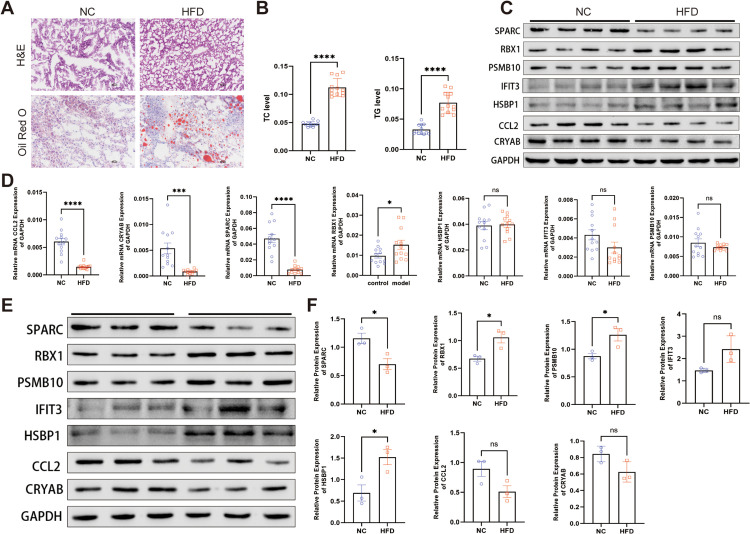
The relative expressions of optimal FGs were confirmed by qRT-PCR and WBs in vivo and in vitro. (A) Representative H&E-staining, Oil Red O-stained liver sections of HFD and NC mice, n = 4/cohort. (B) Serum TC and TG contents of two cohorts testified per the supplier’s protocols. (C) The expression levels of the seven genes from 16 weeks of normal chow (NC) or HFD-fed mice were determined by WBs (n = 4/cohort). (D) The relative expressions of optimal FGs were confirmed by qRT-PCR. (E-F) The expression levels of the seven genes from fibroblasts were determined by WBs with quantification.

We investigated the comparative expression of seven optimal FGs in vivo. A murine NAFLD model was established by administering a HFD to mice for 16 weeks. The H&E and oil red O staining in Fig 10A indicate the successful model construction. TC and TG levels were notably increased in the HFD cohort (Fig 10B). In comparison to the food of diet mice, RBX1, PSMB10, and IFIT3 expression were markedly elevated in HFD mice, aligning with the bioinformatics analysis. Conversely, CCL2, CRYAB, and SPARC expression were markedly reduced in HFD mice (Fig 10C and 10D). Subsequently, we assessed the relative expression of seven optimal FGs at the protein level in fibroblasts treated with OAPA and BSA. SPARC, CCL2, and CRYAB expression decreased in FX2 after OAPA interference, while PBX1, PAMB10, IFIT3, and HSBP1 expression increased accordingly (Fig 10E and 10F).

## Discussion

Nonalcoholic fatty liver disease has become a growing worldwide health concern due to significant shifts in individuals’ eating habits. In this study, we proceeded with analysis to demonstrate that 4 special subtypes of fibroblasts only appeared in normal livers. From this perspective, 7 HGs were identified by hdWGCNA analysis, including CCL2, CRYAB, HSBP1, IFIT3, PSMB10, RBX1, and SPARC, possibly served as the key genes involved in the pathogenesis of NAFLD. In addition to this, we found some evidence of a strong association between these essential genes and immune infiltration in NAFLD and analyzed the difference in their expression in both internal and external datasets. Furthermore, by targeting the 7 key genes, potential signaling pathways were enriched to help-seeking for how they possibly regulate NAFLD. Eventually, to strengthen the persuasive power of our bioinformatic research, we tested the expression of the 7 genes in vivo and vitro models, which elucidated that most of them do have an expression difference in transcription and protein levels.

Hepatic stellate cells (HSCs) are resident fibroblasts that maintain characteristics of a fibrogenic cell type. This nonparenchymal cell cohort constitutes roughly 5%–8% of all liver cells in a healthy liver [24]. During NASH, the dormant, quiescent HSCs can be stimulated by various factors and transform into myofibroblasts, which display a contractile, proliferative, and fibrogenic phenotype with significant alterations in their gene expression profile [25]. These activated hepatic stellate cells produce a broad spectrum of proinflammatory cytokines, including CCL2, CCL5, IL8, chemokine (C-X-C motif) ligand-12 (CXCL12), and express adhesion molecules, leading to enhanced recruitment and infiltration of immune cells into the liver [26]. Collectively, these observations indicate that HSCs serve a substantial function in creating a conducive environment for NAFLD pathogenesis.

The link between immune infiltration and NAFLD is bidirectional. On one hand, immune cells promote oxidative stress, induce fibrosis, and contribute to liver inflammation and hepatocyte damage through the secretion of proinflammatory cytokines. Conversely, NAFLD itself elicits an immune reaction, encompassing the stimulation of resident liver immune cells and the recruitment of inflammatory cells from the circulation [27]. Our research exhibits that B cells naive, monocytes, activated dendritic cells, and mast cells patients in the disease cohort were markedly higher or lower than those in the control cohort, which pictured a basic landscape of immune infiltration change in NAFLD. It is noted that some of the HGs we discovered are markedly correlated with immune cells in the liver. This may support us in raising presumptions that genes like CCL2, HSBP1, PSMB10, and PBX1 play a key role in immune mechanisms that influence NAFLD pathogenesis if combined with further research.

The connection between NAFLD and CCL2, a C-C chemokine family member, is well-established. In NASH models, Kupffer cells and other hepatic cells produce chemokines like CCL2, triggering substantial infiltration of inflammatory and fibrogenic monocytes to the damaged liver through CCR2-CCL2 interactions [28]. Eliminating CCR2 and CCL2 genetically or inhibiting CCL2 pharmacologically mitigates liver fibrosis in mice [29]. Notably, CCL2 may directly promote steatosis besides inducing monocyte infiltration [30].CRYAB, also termed HspB5 or αB-Crystallin, is a member of the small heat shock protein family. It is an acknowledged anti-apoptotic protein [31], primarily functioning to negatively regulate proapoptotic Bcl-2 family members, Bax, and caspase-3 [32]. Moreover, extracellular CRYAB participates in anti-inflammatory processes linked to immunoregulatory response activation in macrophages via endosomal/phagosome CD14 and Toll-like receptors 1 and 2 (newly identified CRYAB interacting proteins) [33]. HSBP1 has been recognized as a negative modulator of heat shock factor 1 (HSF1) [34], the principal orchestrator of transcriptional reactions to proteotoxic challenges. Mario et al. demonstrated that the FIP200-ATG13 subcomplex could negatively control HSBP1’s pro-picornaviral role, thus acting as an anti-picornaviral factor [34]. Notably, these findings aligned with our detected pathways, which were enriched in apoptosis, pentose phosphate pathway, proteasome, and ribosome. The interferon‐inducible protein with tetrapeptide repeats 3 (IFIT3) is a crucial member of both the IFIT family and interferon‐stimulated gene family. It plays a biological role in innate immune responses to bacterial and viral infections [35].The immunoproteasome PSMB10 has been demonstrated to have a crucial function in modulating immune responses, oxidative stress, and maintaining cellular protein homeostasis. A thorough understanding of PSMB10’s role in NAFLD is lacking [36]. Research indicates that Rbx1 is involved in mitochondrial ubiquitination in hepatocytes, whose morphological changes may be implicated in NAFLD [37].The SPARC (secreted protein acidic and cysteine-rich) molecule is a matricellular component that modulates interactions between cells and their adjacent extracellular matrix (ECM). As a known proinflammatory adipokine, SPARC is reportedly involved in low-grade inflammation in adipose tissue (AT), a characteristic of obesity contributing to several metabolic abnormalities, encompassing type 2 diabetes and nonalcoholic disease [38].

In our investigation, we constructed a diagnostic framework exhibiting enhanced precision and responsiveness, surpassing the performance of previously suggested biomarkers. Our approach also offered insights into clinical medication utilization and forecasting drug effectiveness. Nevertheless, its practical applicability in clinical settings requires further validation through further research. Our study comprehensively elucidated the underlying principles and operational mechanisms of the diagnostic model by examining the metabolism, immune responses, and cellular growth and proliferation of the model genes from various angles, thereby furnishing substantial theoretical backing for its implementation in clinical practice.

## Supporting information

S1 Data (XLSX)
